# Use of water turnover method to measure mother’s milk flow in a rat model: Application to dams receiving a low protein diet during gestation and lactation

**DOI:** 10.1371/journal.pone.0180550

**Published:** 2017-07-17

**Authors:** Thomas Sevrin, Marie-Cécile Alexandre-Gouabau, Dominique Darmaun, Antoine Palvadeau, Agnès André, Patrick Nguyen, Khadija Ouguerram, Clair-Yves Boquien

**Affiliations:** 1 UMR PhAN, INRA, CRNH Ouest, Université de Nantes, Nantes, France; 2 Nantes Hospital, CHU Hôtel-Dieu, CRNH Ouest, IMAD, DHU2020, Nantes, France; 3 ONIRIS, Nantes-Atlantic National College of Veterinary Medicine, UNE, Nantes, France; Universidade do Estado do Rio de Janeiro, BRAZIL

## Abstract

Assessment of milk production is of utmost relevance for pediatricians and scientists interested in early life nutrition. The weight-suckle-weight (WSW) method, which consists of weighing babies before and after they suckle their mother, uses the difference in body weight as an estimate of milk intake. However, this is prone to many sources of error. In the current study, we used for the first time the water turnover method and compartmental analysis with deuterated water (D_2_O) as a non-toxic tracer to quantify *in vivo* milk production in a rat model. We assessed the effect of a nutritional intervention presumed to affect milk production, a maternal dietary protein restriction during gestation and lactation, which results in the birth of pups with intrauterine growth restriction. The specific aim of this study was to determine milk production with the body water turnover method in rat dams receiving during gestation and lactation, either a control diet (NP) or an iso-caloric low-protein diet (LP). In NP dams, mass of dam’s total body water, output flow constant from dam to litter (K21) and median milk flow, calculated between days 11 to 14 after pup birth, were 282.1 g, 0.0122 h^-1^ and 3.30 g/h for NP dams, respectively. Maternal dietary protein restriction (-59%) during perinatal period led to a 34% reduction in milk flow (NP versus LP). With the WSW method, milk flow varied from 1.96 g/h to 2.37 g/h between days 11 to 14 for NP dams. The main advantage of the D_2_0 method compared to the WSW method stems from its higher precision, as attested by the narrowest range of measured values of milk flow ([2.90; 3.75] and [0.98; 6.85] g/h, respectively) for NP group. This method could be suitable for testing the effectiveness of candidate galactologue molecules presumed to enhance milk production in the lactating rat model.

## Introduction

The World Health Organization recommends exclusive breastfeeding during the first six months of life because breastfeeding has been shown to be associated with health benefits not only for the infant but for the mother as well [1]. Even though the rate of initiation of breastfeeding is as high as 60% in most high-income countries like in France, exclusive breastfeeding rate plummets long before 6 months of age [2]. The main reasons of the early cessation of breastfeeding are (i) the early return of mothers to work but (ii) perceived insufficient lactation was the reason stated by 62% of mothers who stopped breastfeeding [3–5]. It therefore would be of utmost interest to find innovative ways to enhance lactation in these mothers. For this purpose, candidate molecules have to be found and tested, as a first step, in animal models. Rodent models are indispensable for the detailed study of the long-term impact of mother’s milk on the development and health of offspring until adulthood. It is thus crucial to be able to precisely quantitate the production of maternal milk in rodents, and its modulation by maternal supplementation with candidate molecules.

Traditionally, the method used to estimate the volume of milk ingested by infants is the weight-suckle-weight (WSW) method which consists of weighing babies before and after they suckle their mother, and to use the difference in babies’ weight as an estimate of milk intake. This method has the advantage of being non-invasive, inexpensive, quick, and easy to apply in humans [6] as well as in animals models [7]. Nonetheless, the WSW method lacks precision and accuracy because of the small size of weight changes measured, particularly in rodent models. Moreover, the method requires weighing after each nursing period, and does not take into account the metabolic weight loss of newborns during the suckling period due to water loss into urine, feces and sweat [6, 8]. Deuterated water (D_2_O), a common non-toxic tracer, has been successfully used in women [9, 10] and sows [11] to measure *in vivo* milk production. This method that requires the administration of a dose of labeled water to calculate maternal body water, has been shown to be precise and accurate in large animals such as pigs [11]. In the current study, we used for the first time the water turnover method and compartmental analysis to quantify milk production in a rodent model which is relevant for its short generation time. Such method will be useful to study nutrients or bioactive molecules likely to modulate breastmilk production.

To further assess the adequacy of the approach, we studied a nutritional intervention presumed to affect milk production. We used maternal dietary protein restriction during gestation and lactation, which results in the birth of pups with intrauterine growth restriction [12, 13]. Moreover, low maternal protein intake maintained throughout lactation induces extra-uterine growth restriction [13], which may result from either (a) physiologic modifications related to the low birth weight *per se* [12, 13], (b) changes in milk composition, as shown in our earlier study [14], or (c) a reduction in milk yield, as suggested by BAUTISTA et al. [15] who used the WSW method in a rodent model.

The specific aims of this study were (i) to determine milk production in rat dams by studying body water turnover; and (ii) to apply this method to measure milk production in dams receiving during gestation and lactation, either a control diet or an isocaloric, low-protein diet.

## Materials and methods

### Animal experiments, housing and diets

Animal experiments were performed in accordance with current European regulations regarding the protection of animals used for experimentation, and after approval of the experimental protocol (project 2015112514233593-APAFIS 3127) by the regional ethics committee (CEEA, Comité d’éthique en expérimentation animale—Pays de la Loire, France). Sprague-Dawley female rats purchased from JANVIER LABS (Le Genest-Saint-Isle, France) were placed from the first day of gestation (GD 1) in cages with wood chips in a room maintained between 19 and 22°C, between 35 and 50% of relative humidity, and under a twelve hours day/night cycle (light from 6:00 a.m. to 6:00 p.m.). Animals had access to food and water *ad libitum* and were randomly assigned from GD 1 to two different diets throughout gestation and lactation: a control diet (normal protein diet, NP) with 20 g protein per 100 g of food or an isocaloric low-protein diet (LP) with 8 g protein per 100 g of food. Both NP and LP diets, as previously used [12, 13], were purchased from ABDIET (Woerden, The Netherlands) (see reference [16] for detailed energy and nutrient composition of both experimental diets) and contained 9–22% casein, 55.15–68.17% dextrose, and 4.30–4.30% soya oil, with a total of 327.27–367.40 Kcal/100g, respectively.

Delivery day at day 21 of gestation was considered postnatal day 0 (PND 0) of lactation. As the time of delivery varied between dams, adjustment of litter size was performed at PND 1. Litter size was adjusted to 8 pups per dam with four males and four females to account for gender effects. Mothers’ mass, food consumption, and litters’ mass were measured daily using a scale (precision of 10 mg) from PND 1 to PND 18.

Two experiments were performed: experiment 1 was carried out with the deuterated water method to assess milk production with the 2 different diets (NP, n = 4; LP, n = 5). Experiment 2 was carried out to assess milk production in control NP dams (n = 5) *vs*. LP dams (n = 6) with the conventional WSW method.

### Experiment 1—Water turnover method

#### Deuterium oxide injection and body fluid sampling

We first determined in preliminary experiments, the time required for complete isotope equilibrium throughout maternal body water following intravenous administration of deuterium labeled water. This allowed us to select the time at which blood could be sampled to determine the tracer dilution and accurately calculate maternal total body water. Accurate determination of this value is essential to allow for a precise calculation of milk flow from mother to litters. In this preliminary experiment, we also compare three ways of administering deuterium (intravenous, intraperitoneal or subcutaneous) and we retained the intravenous route for the rapidity of D_2_O dilution.

To calculate the lactation flow we carried out a whole body water turnover study in mothers and pups by following deuterium enrichment time curves in plasma and urine sampled from mothers and pups, respectively. At day 7 of lactation (PND 7), baseline plasma and urine sample was collected from dams and pups, respectively, prior to intravenous administration of D_2_O, to determine baseline body D_2_O abundance. At PND 11, when lactation is well established, mothers received a 5 g.kg^-1^ (5.00±0.08 g.kg^-1^) intravenous injection of deuterated water (99.9 mole % D_2_-enrichment) purchased from SIGMA-ALDRICH (Saint Louis, USA). After dams received isoflurane anesthesia (4% isoflurane in room air for 5 minutes), injections were carried out using 2mL syringes with 0.5X16 mm needles. Syringes of D_2_O were weighed before and after injection with a precision scale of 0.1 mg.

Three-hundred μL of blood were collected from mothers at 3 h, 24 h, 48 h and 72 h after D_2_O injection, and 200 μL of a pool of urine was collected from pups at 24 h, 48 h, 72 h and 96 h after D_2_O injection. Dams’ blood samples were obtained by tail snip after 5 min-isoflurane anesthesia, collected in vacuum tube containing ethylenediamine tetraacetate, and centrifuged at 1050 g and 4°C for 10 min. One hundred and fifty μL of plasma were collected in Eppendorf tubes and stored at -20°C until analysis. Before urine sampling, pups were separated for 30 min from their mothers in order to avoid urination due to maternal stimulation of pups’ bladders. Pups’ lower bellies were stimulated with iced cotton bud and urine was collected and pooled for each litter in Eppendorf tubes by using a Pasteur pipette and stored at -20°C until analysis. The D_2_O enrichment of both dams’ plasma and pups’ urine samples was measured using a Fourier Transform infrared spectrophotometer (FTIR spectrophotometer) Vector 33 from Bruker (Rheinstetten, Germany) [17].

#### Calculation of milk flow

In practice, D_2_O concentration of all mothers’ plasma and pups’ urine samples was determined by FTIR spectrophotometer and then basal D_2_O concentration was deducted.

**Fig 1:**
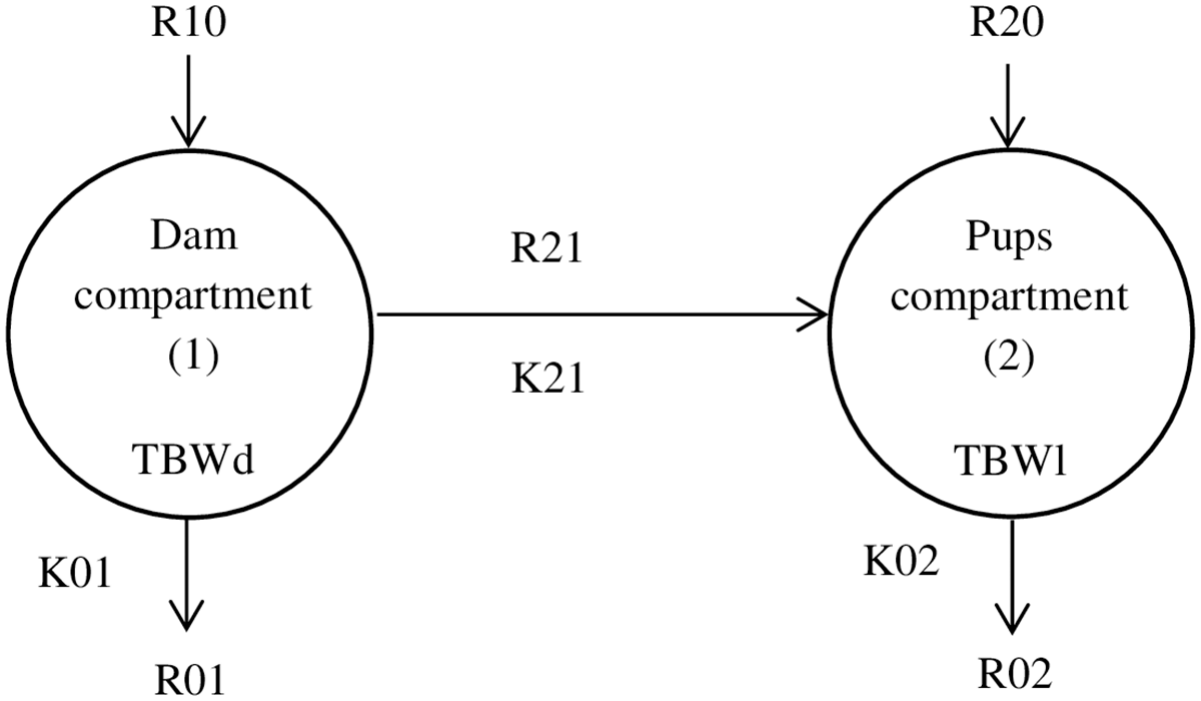
Bi-compartmental model. TBWd, TBWl: Total body water (expressed in g) of dam and litter, respectively; K01 and K02 (expressed in h^-1^) are the output flow constants from dam and litter, respectively; K21 is the output flow constant (expressed in h^-1^) from dam to litter; R10 and R20: Input water flow (expressed in g.h^-1^) into dam and pups, respectively; R01 and R02: Output water flow (expressed in g.h^-1^) from dam and pups, respectively; R21: Water flow from dam to pups (milk) (expressed in g.h^-1^).

Plasma and urine D_2_O concentration data were subjected to compartmental analysis to calculate milk flow between the dam and its litter. We used single-compartment models to describe the dynamic aspects of water metabolism both in mother and its litter related by milk flow which led to a bicompartmental model (Fig 1). The SAAM II program was used to fit the model to the observed tracer data by a weighed-least-squares approach to find the best fit as previously described [18] and to determine the parameters of the model. Implicit in the use of this model is the assumption that each animal remains in steady state with respect to its total body water turnover during the time course of the study. This condition is justified for the total body water of mother compartment as showed by constant body weight (results not shown). We assumed near-steady state for pups’ body water pool as well.

The first and second compartments correspond to the turnover of total body water of mother and litter respectively, resulting from both intestinal absorption and metabolic processes. Fig 1 presents the different absolute production rates of the model: R10 and R20 are the inputs into body of dam and pups, respectively, arising from water drinking and non-dietary water (metabolic water production, respiratory and cutaneous influx of atmospheric water); R01 and R02 are the outputs of water by transpiration, urine, feces, from dam and pups, respectively; R21 is the flow from dam to its litter. R10 is the sum of R01 plus R21 at steady-state. The model has three unknown parameters: (i) K01 and K02 are the output flow constants from dam and litter, respectively; (ii) K21 is the output flow constant from dam to litter.

Mass of Dam’s Total Body Water was calculated by dividing the amount of deuterium oxide injected by the D_2_O value extrapolated from D_2_O concentration curve to the intercept with y axis at time 0. Dam’s Total Body Water (TBWd) expressed in % was calculated by dividing this mass of Body Water by dam’s mass. Flow constants K21, K01 and K02 of the model are calculable directly from the plasma and urine D_2_O enrichment-time curve. The flow from mother to pups R21 (g/h) was calculated as the product of K21 (h^-1^) and the mass of Dam’s Total Body Water (g).

In this model, milk flow was associated to milk production by the dam as well as milk consumption by the litter. Furthermore, values obtained by this method were mean production values throughout the sampling period (PND 11- PND 14).

### Experiment 2—WSW method

Pups were weighed a first time after 1h separation from their mother (P1). Then, they were returned to the mother for 1h suckling, after which they were weighed a second time (P2). Mean of milk pups’ consumption was estimated by the mean difference in weight P2-P1 (dP) for each pup, and was divided by suckling time (dP/t). Dam’s milk production was determined by multiplying the mean milk consumption by the number of pups per litter (8). With this method, results were recorded each day from PND 11 to PND 14.

### Statistics

Non-parametric test was used for kinetic parameters obtained from water turnover study and growth parameters. Mann-Whitney tests (GraphPad Prism, version 6.0) were used to analyze differences between the 2 regimens (NP, LP) for birth weight, pups’ daily mass gain between PND 1 and PND 18, dam’s food intake from PND8 to PND16, dams’ TBW, and milk production. Data obtained using kinetic study and growth are expressed in median and upper and lower quartiles due to small sample sizes. Regarding the WSW method, due to larger sample sizes, we assumed Gaussian distribution, and used unpaired Student tests with Welch’s correction (we did not assume equal standard-deviations) to compare milk production between the 2 regimens (software GraphPad Prism, version 6.0). Mean values and standard deviations are therefore reported. Statistical significance was set to a confidence level of p < 0.05.

## Results

### Pup growth (Experiment 1)

Growth was followed in experiment 1 on 4 and 5 litters in the NP and LP groups, respectively. At PND1, median dam mass was 337.7 g in NP group and 308.3 g in LP group. Dam’s mass variation between PND 1 and PND 18 was lower than 10% in the two groups. Between PND8 and PND16, average food intake was 0.14 ± 0.02 g/g body weight/d for NP dam and 0.13 ± 0.02 g/g body weight/d for LP dam without any significant difference (p = 0.063). Taking into account the composition of both diets [16], maternal protein intake was 59% lower in LP group than in NP group while energy intakes were similar, arguing more in favor of a deficiency in protein rather than in energy intake in this model.

**Fig 2:**
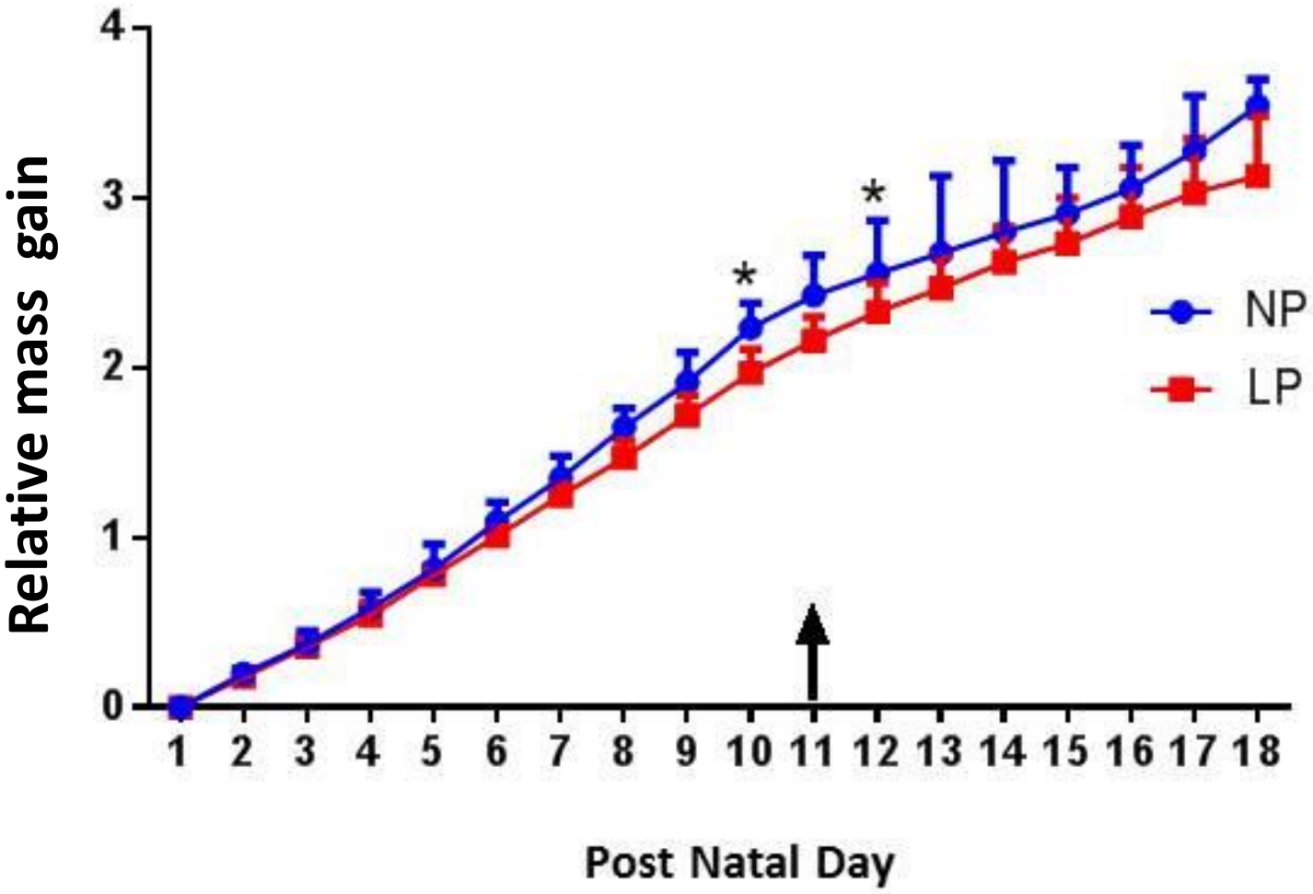
Relative mass gain (median ± range) of pups from PND 1 to PND 18 in the NP and LP groups. NP, n = 4; LP, n = 5. The arrow represents the day of deuterium oxide injection. Differences between nutritional groups were analyzed at each day using a Mann-Whitney test: * P-value<0.05.

Pup body weight at PND 1 was not different between groups (7.59 g and 7.33 g, in NP and LP respectively). Relative mass gain (RMG) was calculated from PND 1 to PND 18 (Fig 2). To calculate pups’ RMG, birth weight was subtracted from daily weight and the obtained value was divided by birth weight. RMG showed a trend to be lower in LP compared to NP (p<0.08) at PND 10 and 12. At the end of the period (PND 18), no significant difference was shown between NP and LP RMG (3.54 and 3.13, respectively).

### Milk production measurement with the deuterium oxide method (Experiment 1)

#### Maternal total body water (TBWd)

**Fig 3:**
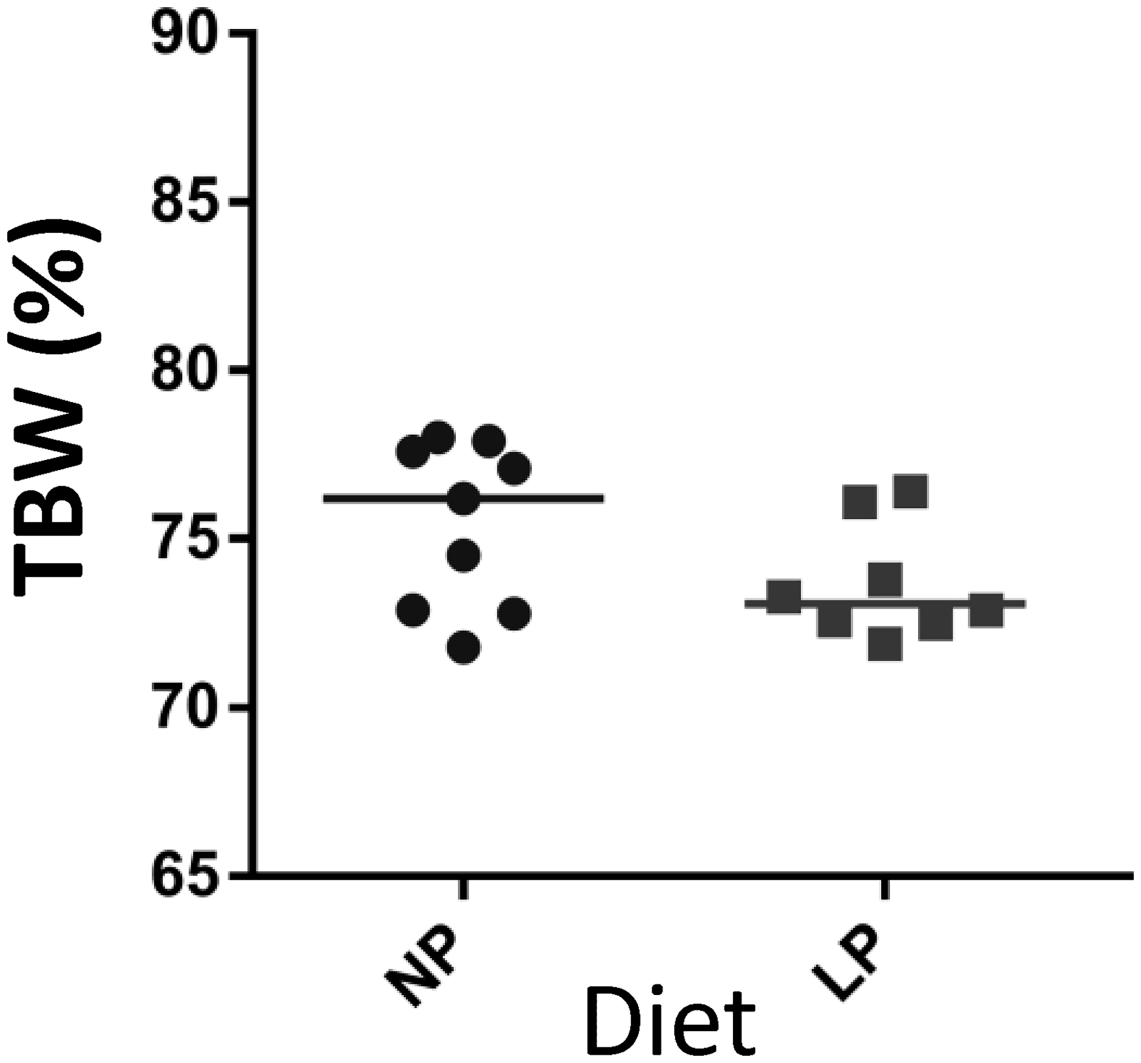
Distribution of maternal total body water in the NP and LP groups. NP, n = 4; LP, n = 5. Median differences between groups were been analyzed by a Mann-Whitney test.

A representative D_2_O concentration kinetic curve in mother’s plasma is shown in S1 Fig. Mass of Dam Total Body Water was calculated as explained in the Methods section by dividing the amount of deuterium oxide injected by D_2_O concentration at time zero obtained from extrapolating curve and data are shown in Fig 3. Mass of total body water was significantly higher in NP dams (282.1 g) than in LP dams (236.4 g) (p = 0.032) on the PND 11 to PND 14 lactation period. We found median Dam Total Body Water (TBWd) values of 76.9% [72.8%–77.9%] of body weight for NP group and 72.9% [71.9%–76.4%] for LP group with no significant difference between groups. The median value of dams TBWd for both groups was 76.1%.

#### Milk flow

**Fig 4:**
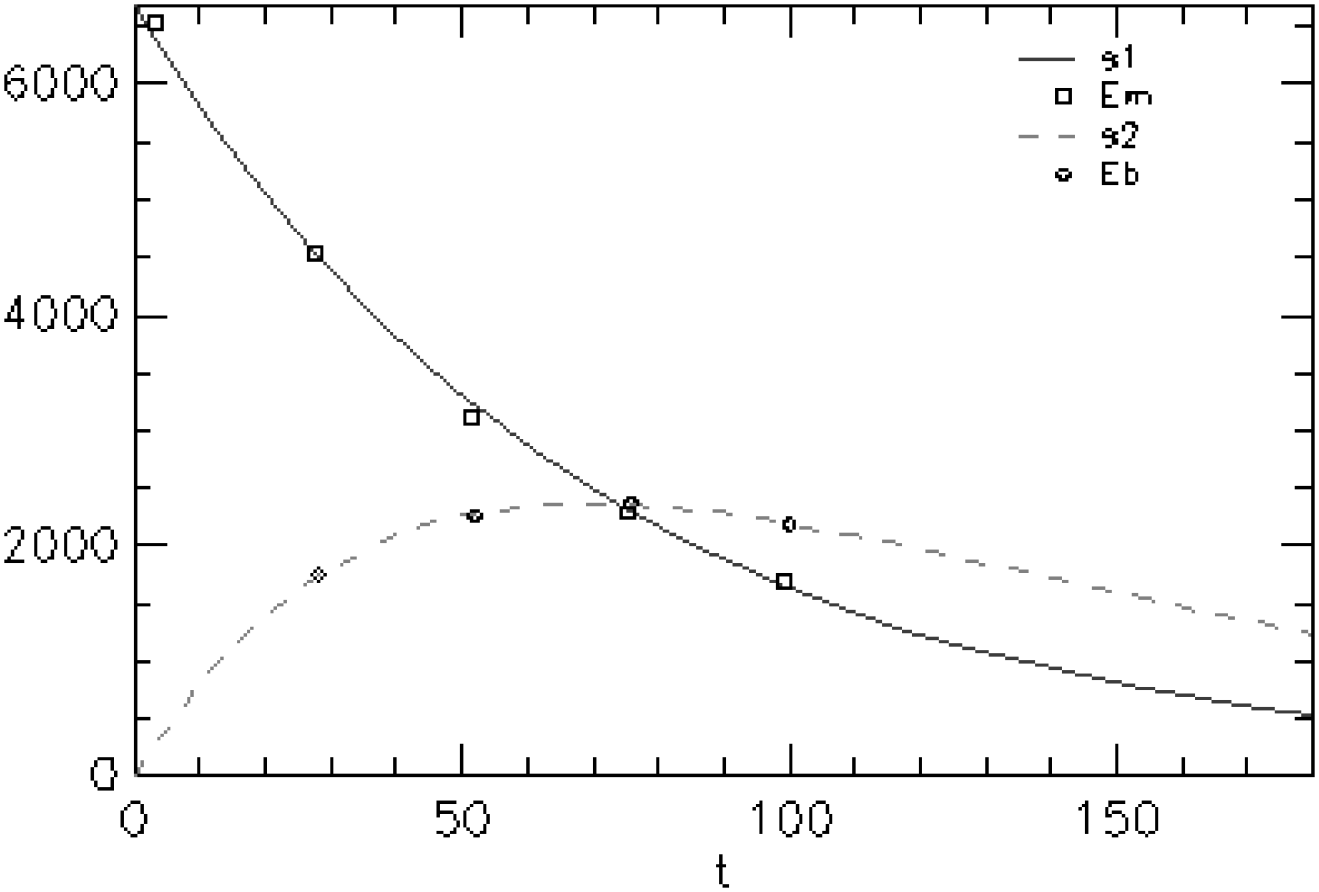
Time course of isotopic enrichment in mother plasma and litter urine after injection of deuterated water to a representative mother (group NP). Points represent data values. Lines represent curves fitted using the model of Fig 1.

D_2_O concentration kinetic curves in mother’s plasma and pups’ urine are shown in Fig 4. Model fitted lines and experimental points showed close agreement. Output flow constant from dam to its litter (K21) was 0.0122 h^-1^ for NP dams (Table 1). K21 had a strong trend to be higher in NP than LP group (p = 0.06). Median variation coefficient was maximum 5.1% on the PND 11 to PND 14 lactation period (Table 1).

**Table 1:** Output flow constant (K21) obtained after fitting with SAAM II software and milk flow per dam in the NP and LP groups in the period ranging from PND 11 to PND 14. Difference between groups was analyzed with a Mann-Whitney test: * P-value < 0.05.

Group		NP	LP
n (dams)		4	5
**Output flow constant (K21)**	Median K21 (h^-1^)	0.01223	0.00980
	Median standard deviation	0.00036	0.00047
	Median variation coefficient	3.0%	5.1%
**Milk flow**	Median milk flow (g/h)	3.30	2.18*
	Min, Max	(2.90; 3.75)	(1.73; 2.55)

Milk flow between the dam and its litter was obtained by multiplying values of K21 by TBWd. Values obtained were median values between PND 11 to PND 14 period and were expressed in g/h. Milk flow was 3.30 g/h [2.90; 3.75] for NP dams and 2.18 g/h [1.73; 2.55] for LP dams (Table 1). Milk flow was significantly higher in NP than LP (p = 0.016) dams. Maternal dietary protein restriction during perinatal period led to a 34% reduction in milk flow (NP versus LP).

### Milk production measurement with the WSW method (Experiment 2)

The WSW method was applied in experiment 2 with NP and LP dams (n = 5 and n = 6, respectively). Results were obtained for each day in g/h from PND 11 through PND 14 (Table 2). Milk flow varied from 1.96±1.71 g/h at PND 12 to 2.37±1.82 g/h at PND 14 for NP dams, versus 1.41±2.61 g/h at PND 11 to 1.28±0.66 g/h at PND 14 for LP dams. Mean milk production was found to be significantly lower only on PND 14 (P<0.001). We observed that variations seemed to be larger in NP group than in LP group, as attested by the ranges on measured values of milk flow between PND 12 and PND 14 ([0.98; 6.85] g/h for NP group and [0.63; 3.95] g/h for LP group, respectively). No significant difference was observed at PND 12 and PND 13 between milk flow with the D_2_O method and with the WSW method for NP and LP dams except at PND 12 for NP dams (p = 0.014).

**Table 2:** Milk production obtained by the weight-suckle-weight method from PND 11 to PND 14 in NP and LP groups.

Group		NP	LP
n (pups)		40	48
**Milk production per dam (g/h)**	PND 11	nd	1.41±2.61
	PND 12	1.96±1.71	1.54±1.19
	PND 13	2.50±3.25	1.87±1.10
	PND 14	2.37±1.82	1.28±0.66*

Data are means ± SD. Differences between groups were analyzed with Student test.

*P-value<0.001 vs NP.

nd: not determined

## Discussion

To the best of our knowledge, the current study is first to report on the use of deuterium oxide and compartment modeling to assess rates of milk production in rats, and to explore the effect of manipulating maternal diet on milk production in lactating dams using such method. Our results suggest that this method is usable in rodents to determine milk production and its modulation by nutrients. By using this method, we showed that a reduction in maternal protein intake (-59%) during both gestation and lactation period decreases milk flow (-34%).

Our bi-compartmental model is based on the hypothesis that at least the dam’s compartment is at steady state. This is probably true because the experiment was performed at a time when dams had a stable body weight and lactation was well established so that the variation in TBWd should be minimal. This is less likely to apply for pups since they are growing between PND 11 and PND 14. As the calculations for the determination of milk flow only use parameters from mother compartment, data were analyzed using a steady state model. We believe that this model is consistent as i) the model fits properly the D_2_O enrichment data; ii) coefficients of variation are below 5% for most calculated parameter values; and iii) values obtained for TBWd and of milk flow are in accordance with data already published (see below).

As the deuterium oxide method and the WSW method could not feasibly be applied in the very same individual dams in order to limit animal stress, we applied the whole body water turnover approach in separate, comparable groups of lactating dams submitted to the same dietary manipulation. With the deuterated water method, we found median milk production values of 3.30 g/h, and 2.18 g/h for NP and LP dams, respectively, on the period ranging from PND 11 to PND 14. Our results obtained using D_2_O method are not significantly different from those we found using the WSW method, particularly at PND 13 for both NP and LP groups (2.50 g/h and 1.87 g/h, respectively), but with an average overestimation of 24%. Similar findings were obtained in a pig model by THEIL et al. [7], who reported an average underestimation of 12.7% of milk production (max 21.3% on day 11 of lactation) with the WSW method compared to the D_2_O method. In the current study, the D_2_O method was, however, more precise than the WSW method, as attested by the lowest range of measured values of milk flow ([2.90; 3.75] and [0.98; 6.85] g/h, respectively) for NP group. This is partly due to the high precision of the FTIR spectrophotometer producing only a ≈ 1% error in the determination of deuterium oxide enrichment. The high variation coefficient obtained with the WSW method may be accounted for by the unmeasured, inevitable weight loss as urine, feces and sweat occurring during suckling period [8] but, the most likely explanation is the minuscule body weight changes to be measured in pups over a single hour of suckling time as suggested earlier [6].

We found median TBWd of 76.9% and 72.9% for NP and LP dams, respectively, without any significant difference between groups. Our results suggest that perinatal diet did not affect the dam’s total body water. The ‘gold standard’ method to measure TBW is desiccation. FOY and SCHEINDEN [19] found an average of 65.0% of body water in albinos rats using this method, a value 15% below our measured values. Our overestimation of TBWd could be explained by: (i) the short duration of the kinetic study (additional sampling after 96 hours could be useful to obtain more accurate values, but we wanted to minimize sampling time for ethical reasons); (ii) the exchange of deuterium with labile hydrogen of protein and other body components [20] in the isotope dilution method; (iii) the fact that our dams were lactating which is associated with a 5% increase in TBW [21]. This overestimation of TBWd unavoidably leads to a slight overestimation of milk production, compared with the WSW method.

In their study, BAUTISTA et al. [15] used the WSW technique to calculate milk intake of pups born from control dams or dams fed a protein restricted diet; they deduced milk production by multiplying the individual pup’s milk intake (in g/h) by the number of pups per litter. The authors estimated the milk production between 3.24 g/h and 3.78 g/h for NP dams and between 1.84 g/h and 2.14 g/h for LP dams at PND 14 for large litters (12 to 14, according to the authors). These values are closer to those found using the D_2_O method (NP = 3.30 g/h and LP = 2.18 g/h) and higher than those obtained with WSW method (NP = 2.37 g/h and LP = 1.28 g/h at PND 14) in our study. Several factors may account for the underestimation of milk production with the WSW method in our study. First the separation time of the pups from the dams was 4h in BAUTISTA et al. [15] study versus 1h in the present study. A longer starvation likely leads to a higher milk intake by the pups when they are allowed to suckle, but may also exacerbate offspring’s stress. On the other hand, the larger the litter, the higher the milk production [22]. The lower milk production of our dams determined using the WSW method could be explained by the smaller litter size (8 versus 12 to 14 in BAUTISTA et al. [15] study). Altogether, these findings are consistent with the view that D_2_O method can yield reliable measurement of milk production in a rodent model. Contrary to the WSW method that can provide a daily milk production value, the D_2_O method only yields an average milk flow value over several days of lactation, which may be more representative of the overall period of lactation, and allow to smooth putative day-to-day fluctuations over the course of lactation.

The deuterium oxide method only requires small blood sample volumes for D_2_O measurements, and does not require long separation of pups from their mother (30 min *vs*. 60 min in the WSW method in our study, or *vs*. 4h in BAUTISTA al. [15], respectively) which is stressful and stress could, in turn, introduce bias. Although we are aware that in the D_2_O method, blood sampling from the dams after isoflurane anesthesia at PND7 and between PND11 and PND14 can induce stress, the degree of stress likely was mild since it had no apparent impact on pup growth. Moreover, isoflurane exposure was shown to have the least effect, compared to short-term exposure to other anesthetic agents such as diethyl ether or CO_2_, on plasma cortisone, glucose and insulin levels in male rats weighing 180–210 g, suggesting a low impact of isoflurane on metabolic status in male adult rats [23]. Regarding dam’s food intake, we observed a substantial daily decrease (max 37%) in food intake after D_2_O injection to the dams, only in a few LP dams (3 out of 5), not in NP dams. This decrease was always reversible and did not last more than 1 or 2 days maximum. This suggests that the reduction in food intake was likely due to dietary protein *per se* in LP dams rather than to the D_2_O method.

Due to its higher precision compared with the WSW method, the D_2_O method may also be more appropriate to study the effect of dietary or pharmacological manipulation of lactating dam on its milk production. Indeed, with the D_2_O method, milk production was 34% lower in LP dams, compared with control dams (P = 0.016) whereas the difference failed to reach statistical significance with the WSW method, except at PND 14 (P = 0.0008) during the peak of lactation. The current results obtained with the D_2_O method confirm that perinatal protein restriction results in a decrease in milk yield as suggested by BAUTISTA et al. [15]. Indeed MORETTO et al. [24] have previously shown a decrease in the development of mammary gland and prolactin secretion, both involved in milk synthesis [25], in Wistar dams in response to low protein (6%) diet of dams provided through pregnancy and lactation. We have also to consider that the mass of total body water was lower in LP dams compared to NP dams, suggesting that the pool of water available for milk production is lower in LP dam.

The high precision of our D_2_O method compared to the WSW method resulted from (i) a high precision in the quantity of D_2_0 injected in intravenous route, by weighing the syringe containing D_2_0 solution before and after injection with a precision of 0.1 mg; (ii) sufficient rise in dams’ plasma D_2_O enrichment with an injection of 5 g.kg^-1^ of deuterium water compared to the natural abundance of D_2_0 at 155 ppm under baseline condition [26]; (iii) a higher flexibility in biological sampling in offspring as non-invasive urine sampling could be performed on separated pups and then pooled only if necessary; and (iv) loss of water by pups by urine, feces, and evaporation during suckling could be a non-negligible source of error in the WSW method and not in the D_2_O method. With the WSW method, the whole litter, rather than separate pups were weighed for a better quality of mass measurement.

## Conclusion

We found that in lactating rodents, the D_2_O dilution method yields milk flow values close to those found using the traditional WSW method, and seems to be more precise. The main advantage of the D_2_0 method compared to the WSW method stems from its higher precision, as attested by the narrowest range of measured values. This results in smoothing the day-to-day fluctuation in milk flow determined over the course of lactation, and allows for the use of a smaller number of dams to detect changes in milk flow. This method could be suitable for the detection of relatively small changes in milk production due to physiological alterations in the lactating mother, and for testing the effectiveness of candidate galactologue molecules presumed to enhance milk production in the lactating rat model.

## Supporting information

S1 Fig Time course of D_2_O elimination in maternal plasma (linear plot and semi-log plot) in a representative dam. (TIF)

S1 File Data relative to experiment 1 (D_2_O method) (Pages 1 to 4) and experiment 2 (WSW method) (Page 5). Pages 1 and 2: Dam’s mass and food intake for NP and LP groups, respectively; Page 3: pup’s mass and relative mass gain (RMG) for NP and LP groups; Page 4: Water volume (g), K(2,1), and milk flow (g/h) for NP and LP groups; Page 5: Milk flow (g/h) between PND 11 and PND 14.
(PDF)

## Abbreviations

D: Deuterium
D_2_O: Deuterium Oxide
GD: Gestational Day
LP: Low Protein
NP: Normo Protein
PND: Post Natal Day
RMG: Relative Mass Gain
TBW: Total Body Water (of dam (TBWd) and of litter (TBWl))
WSW: Weight-Suckle-Weight
