# Supplementary figures and images for: Use of water turnover method to measure mother’s milk flow in a rat model: Application to dams receiving a low protein diet during gestation and lactation
Source: PLoS One. 2017 Jul 17;12(7):e0180550. doi: 10.1371/journal.pone.0180550 (PMC5513591; doi:10.1371/journal.pone.0180550)

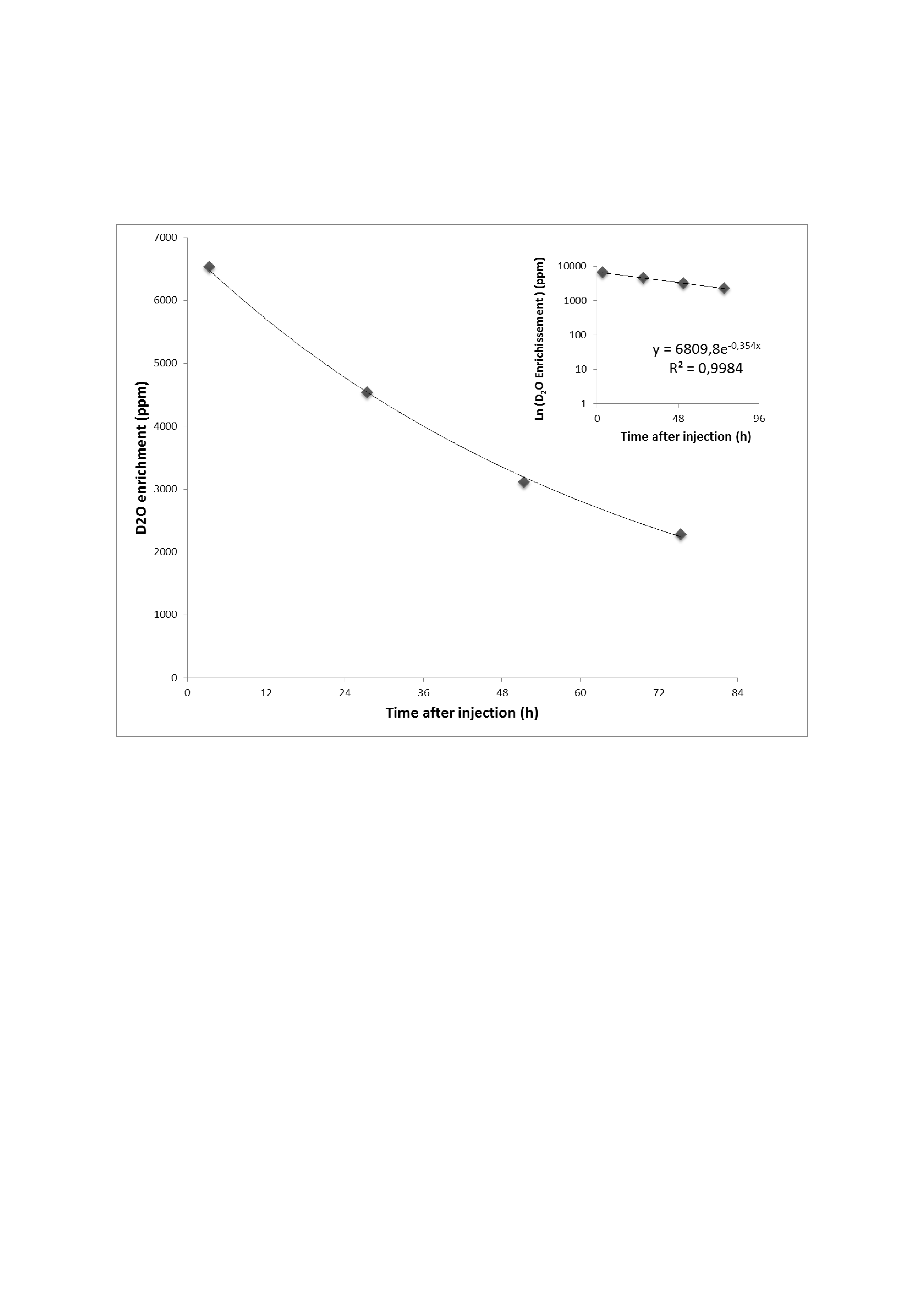

Supplement: S1 Fig — (TIF) [file pone.0180550.s001.tif]
