# Supplementary material for: Use of water turnover method to measure mother’s milk flow in a rat model: Application to dams receiving a low protein diet during gestation and lactation
Source: PLoS One. 2017 Jul 17;12(7):e0180550. doi: 10.1371/journal.pone.0180550 (PMC5513591; doi:10.1371/journal.pone.0180550)
Supplement: S1 File — Pages 1 and 2: Dam’s mass and food intake for NP and LP groups, respectively; Page 3: pup’s mass and relative mass gain (RMG) for NP and LP groups; Page 4: Water volume (g), K(2,1), and milk flow (g/h) for NP and LP groups; Page 5: Milk flow (g/h) between PND 11 and PND 14. (PDF) [file pone.0180550.s002.pdf]

Exp 1	day after birth	Dam's mass
NP		Median
	1	337,7
	18	360,3

PN day	Food Intake (g/g/day)			
	NP1	NP2	NP3	NP4
1				
2				
3				
4				
5				
6				
7				
8	0,12	0,10	0,15	0,14
9	0,14	0,13	0,14	0,14
10	0,11	0,15	0,17	0,13
11	0,14	0,12	0,15	0,15
12	0,12	0,11	0,14	0,13
13	0,13	0,13	0,14	0,14
14	0,12	0,15	0,17	0,16
15	0,15	0,12	0,15	0,13
16	0,15	0,15	0,14	0,15
17				
18				

Food Intake (g/g/day)	
Average	SD
0,14	0,02

LP	day after birth	Dam's mass Median
	1	308,3
	18	299,8

Food Intake (g/g/day)

PN day	LP1	LP2	LP3	LP4	LP5
1					
2					
3					
4					
5					
6					
7					
8	0,15	0,14	0,12	0,14	0,12
9	0,14	0,15	0,12	0,12	0,13
10	0,13	0,13	0,15	0,13	0,14
11	0,13	0,12	0,11	0,14	0,14
12	0,14	0,14	0,09	0,11	0,11
13	0,12	0,13	0,10	0,10	0,12
14	0,13	0,12	0,14	0,09	0,07
15	0,15	0,16	0,11	0,15	0,15
16	0,16	0,14	0,16	0,15	0,12
17					
18					

Food Intake (g/g/day)

Average	SD
0,13	0,02

PN day	median Pups' mass (g)	
	NP	LP
1	7,59	7,33
2	9,06	8,63
3	10,38	10,11
4	12,08	11,46
5	13,80	13,22
6	15,74	14,22
7	17,67	16,50
8	19,90	17,36
9	22,25	19,58
10	24,53	21,42
11	25,98	23,09
12	26,96	24,42
13	28,18	25,45
14	29,26	25,84
15	30,07	26,99
16	30,80	28,25
17	32,04	29,37
18	34,07	30,27

PN day	median RMG	
	NP	LP
1		
2	0,20	0,18
3	0,37	0,36
4	0,59	0,54
5	0,82	0,78
6	1,10	1,01
7	1,35	1,25
8	1,65	1,47
9	1,92	1,72
10	2,23	1,97
11	2,42	2,16
12	2,55	2,33
13	2,68	2,47
14	2,80	2,62
15	2,91	2,73
16	3,06	2,89
17	3,27	3,03
18	3,54	3,13

D2O method				
	NP			
dam	NP1	NP2	NP3	NP4
Water volume (g)	294,8	296,0	237,2	269,3
median	282,1			
K (2,1) SAAM II (h^{-1})	0,01013	0,01224	0,01222	0,01392
median	0,01223			
SD k(2,1)	0,00085	0,00042	0,00030	0,00013
median SD	0,00036			
median CV	3,0%			
Flow (g/h)	2,99	3,62	2,90	3,75
median	3,30			
TBW dam	72,8%	76,2%	77,6%	77,9%
median	76,9%			

D2O method					
	LP				
dam	LP1	LP2	LP3	LP4	LP5
Water volume (g)	237,6	210,0	222,0	236,7	236,4
median	236,4				
K (2,1) SAAM II (h^{-1})	0,00747	0,00822	0,00980	0,01077	0,01054
median	0,00980				
SD k(2,1)	0,00117	0,00047	0,00050	0,00037	0,00019
median SD	0,00047				
median CV	5,1%				
Flow (g/h)	1,77	1,73	2,18	2,55	2,49
median	2,18				
TBW dam	71,9%	72,9%	72,6%	76,4%	76,1%
median	72,9%				

Exp 2	Milk flow (g/h) - WSW			
NP	PND11	PND12	PND13	PND14
Average (n=40)		1,96	2,50	2,37
SD		1,71	3,25	1,82

	Milk flow (g/h) - WSW		
median (n=8)	PND12	PND13	PND14
NP5	2,36	1,54	2,04
NP6	1,22	0,98	2,64
NP7	1,76	1,48	2,49
NP8	1,81	2,44	1,23
NP9	2,78	6,85	2,40

min	max
0,98	6,85

	Milk flow (g/h) - WSW			
LP	PND11	PND12	PND13	PND14
Average (n=40)	1,41	1,54	1,87	1,28
SD	2,61	1,19	1,10	0,66

	Milk flow (g/h) - WSW		
median (n=8)	PND12	PND13	PND14
LP6	1,31	1,99	1,01
LP7	1,52	1,04	1,28
LP8	0,63	1,04	1,34
LP9	3,80	3,95	1,38
LP10	1,13	1,97	0,74
LP11	0,93	1,42	1,93

min	max
0,63	3,95
